# 2-Aminoimidazoles Inhibit *Mycobacterium abscessus* Biofilms in a Zinc-Dependent Manner

**DOI:** 10.3390/ijms23062950

**Published:** 2022-03-09

**Authors:** Juan M. Belardinelli, Wei Li, Kevin H. Martin, Michael J. Zeiler, Elena Lian, Charlotte Avanzi, Crystal J. Wiersma, Tuan Vu Nguyen, Bhanupriya Angala, Vinicius C. N. de Moura, Victoria Jones, Bradley R. Borlee, Christian Melander, Mary Jackson

**Affiliations:** 1 Mycobacteria Research Laboratories, Department of Microbiology, Immunology and Pathology, Colorado State University, Fort Collins, CO 80523, USA; juan.belardinelli@colostate.edu (J.M.B.); wei.li@colostate.edu (W.L.); elian@rams.colostate.edu (E.L.); charlotte.avanzi@colostate.edu (C.A.); crystal.wiersma@colostate.edu (C.J.W.); bhanupriya.angala@colostate.edu (B.A.); caladov@njhealth.org (V.C.N.d.M.); vickijns@gmail.com (V.J.); 2 Department of Microbiology, Immunology and Pathology, Colorado State University, Fort Collins, CO 80523, USA; kevhmartin@gmail.com (K.H.M.); brad.borlee@colostate.edu (B.R.B.); 3 Department of Chemistry and Biochemistry, University of Notre Dame, Notre Dame, IN 46556, USA; mzeiler@nd.edu (M.J.Z.); cmelande@nd.edu (C.M.); 4 Department of Chemistry, North Carolina State University, Raleigh, NC 27607, USA; tnnguyen@ncsu.edu

**Keywords:** *Mycobacterium abscessus*, nontuberculous mycobacteria, biofilm, 2-aminoimidazoles, zinc

## Abstract

Biofilm growth is thought to be a significant obstacle to the successful treatment of *Mycobacterium abscessus* infections. A search for agents capable of inhibiting *M. abscessus* biofilms led to our interest in 2-aminoimidazoles and related scaffolds, which have proven to display antibiofilm properties against a number of Gram-negative and Gram-positive bacteria, including *Mycobacterium tuberculosis* and *Mycobacterium smegmatis*. The screening of a library of 30 compounds led to the identification of a compound, AB-2-29, which inhibits the formation of *M. abscessus* biofilms with an IC_50_ (the concentration required to inhibit 50% of biofilm formation) in the range of 12.5 to 25 μM. Interestingly, AB-2-29 appears to chelate zinc, and its antibiofilm activity is potentiated by the addition of zinc to the culture medium. Preliminary mechanistic studies indicate that AB-2-29 acts through a distinct mechanism from those reported to date for 2-aminoimidazole compounds.

## 1. Introduction

*Mycobacterium abscessus* subspecies *abscessus* (*Mabs*), *massiliense* (*Mmas*) and *bolletii* (*Mbol*) form a group of opportunistic, rapidly growing mycobacteria that can cause an array of clinical diseases in humans, including lung, skin and soft tissue, central nervous system and disseminated infections. In recent years, the prevalence of pulmonary infections caused by *M. abscessus*, particularly in susceptible individuals with structural or functional lung conditions such as cystic fibrosis (CF), chronic obstructive pulmonary disease (COPD) and bronchiectasis, has been increasing at an alarming rate [1,2,3]. Treatment for *M. abscessus* pulmonary disease as recommended by the American Thoracic Society and the British Thoracic Society is largely empirical [4,5] and consists of 12–24 months of chemotherapy with a minimum of three antibiotics that lack bactericidal activity and are associated with significant adverse effects [6,7,8]. Despite aggressive chemotherapy, treatment outcomes remain poor. *M. abscessus* bacteria are indeed the most antibiotic-resistant and antibiotic-tolerant nontuberculous mycobacteria (NTM) owing to their highly impermeable cell envelope and the variety of efflux pumps and drug- and drug-target-modifying enzymes encoded within their genomes [8,9,10]. Further compounding this problem is the propensity of *M. abscessus* to form biofilms [11] and the clinical evidence for *M. abscessus* biofilm formation within the airways and lung cavity of human patients [12,13,14]. The presence of biofilms, where bacilli are not only shielded from the effect of antibiotics but may also persist in a drug-tolerant state, may help explain why *M. abscessus* lung infections are usually incurable with antibiotic therapy alone and why adjunctive surgical resection of cavities may improve treatment outcome [4].

With the premise that agents capable of inhibiting *M. abscessus* biofilm formation and/or of dispersing established *M. abscessus* biofilms may potentiate the activity of antibiotics used in combination, our attention turned to 2-aminoimidazoles (2-AI). The decision to study 2-AIs was based upon previous studies by the Melander laboratory and others that showed that the 2-AI class of small molecules and related scaffolds (2-aminopyrimidines (2-AP), 2-aminobenzimidazoles (2-ABI), 2-aminoquinazolines and 2-AI-containing meridianin analogs) display broad-spectrum biofilm inhibition and dispersion activity against Gram-negative and Gram-positive bacteria, including *Mycobacterium tuberculosis* and the nontuberculous *Mycobacterium* species, *M. smegmatis* [15,16,17,18,19,20,21,22,23,24]. Importantly, some 2-AI and 2-ABI compounds demonstrated the ability to sensitize *M. tuberculosis* and *M. smegmatis* to isoniazid, rifampicin and β-lactams [15,19,23,25].

We here identify a series of 2-AI compounds with the ability to inhibit the formation of *M. abscessus* biofilms with IC_50_s (the concentration required to inhibit 50% of biofilm formation) in the range of 12.5 to 25 μM. Interestingly, the lead compound of this series, AB-2-29, is a zinc chelator whose antibiofilm activity is enhanced in the presence of zinc.

## 2. Results

### 2.1. Inhibition of M. abscessus Biofilm Formation by 2-AI Compounds

To determine whether 2-AI-based small-molecule compounds and related scaffolds inhibited the formation of *M. abscessus* biofilms, we screened a library of 30 compounds, including twenty-six 2-AI analogs, one 2-AP compound (EL-05-047) and three meridianin analogs (7.079; 7.025 and 8.001), of which two contained the 2-AI moiety (Table 1). The screening was conducted using the *Mmas* reference strain CIP 108297 grown as submerged biofilms on poly-D-lysine-coated plates in chemically defined synthetic CF medium (SCFM) as previously described [11]. Recent studies from our laboratory have indeed shown that SCFM closely mimics the nutritional conditions encountered and metabolic adaptation undergone by *M. abscessus* grown in actual CF sputum [26], making this model more relevant to infection than other models based on laboratory media.

**Figure 1:**
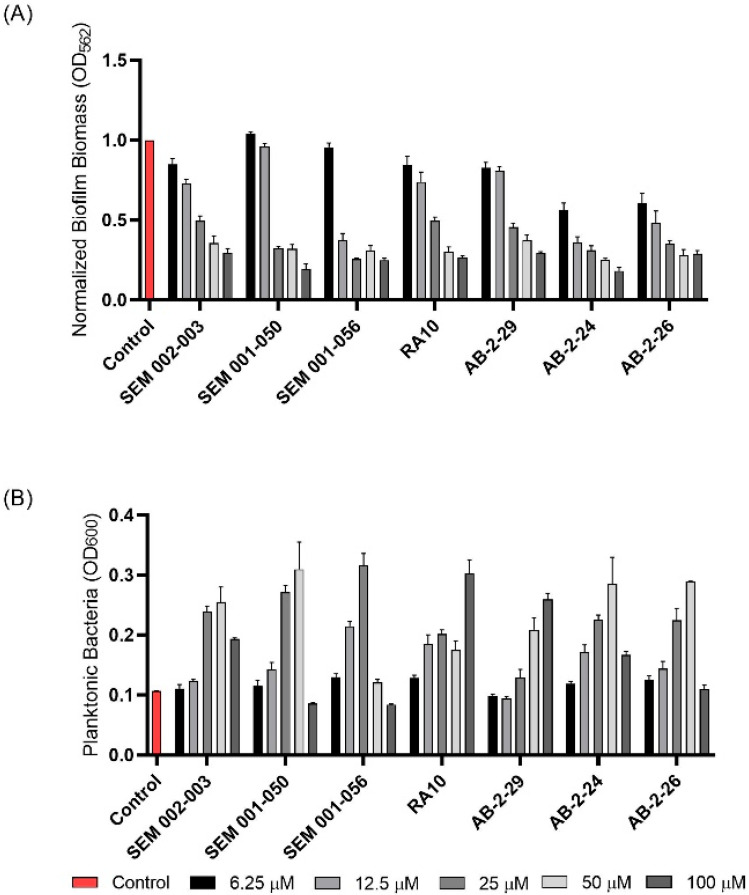
**Effect of 2-aminoimidazoles on *M. abscessus* biofilm formation**. (**A**) Biofilm formation of 2-AI-treated *Mmas* CIP 108297 cultures after 5 days of growth in SCFM medium in poly-D-lysine-coated microplates as determined by crystal violet staining. The compounds were added to the culture medium at the indicated concentrations on the first day and maintained throughout the duration of the experiment. The control corresponds to DMSO diluent (0.2% final concentration) without any added 2-AI compound. (**B**) In parallel, the turbidity of planktonic bacteria released in the medium was assessed spectrophotometrically at 600 nm. Inhibition of biofilm formation correlates with an increase in planktonically growing bacteria in the wells. Decreases in both biofilm and planktonic growth are indicative of the inhibitors having reached their MIC values. The results presented are the means (±SD) of quadruplicate wells and are representative of at least two independent experiments.

Whereas the 2-AP compound and the meridianin analogs failed to show any activity against *Mmas* CIP 108297 biofilms, twelve 2-AI compounds were found to inhibit biofilm formation in a dose-dependent manner and with IC_50_ values at least 2- to 4-fold below their measured MIC in the same medium (Table 1; Figure 1). A subset of these compounds (SEM-002-003; SEM-001-056; SEM-001-050; AB-2-24; AB-2-26, AB-2-29) was retested against other reference and clinical *M. abscessus* isolates encompassing the subspecies *Mabs* and *Mmas* with similar results (Table 1). Subsequent analyses focused on compound AB-2-29, which displayed an IC_50_ less than 4-fold its MIC value. Within the same range of concentrations (6.25 to 50 μM), however, AB-2-29 was not able to disperse pre-established biofilms (Figure S1).

### 2.2. Preliminary Investigations into the Mechanism of Biofilm Inhibition by AB-2-29 in M. abscessus

Three main mechanisms of action have thus far been associated with 2-AI compounds and related scaffolds in Gram-positive and Gram-negative bacteria. First is their ability to interfere with two-component signaling systems, resulting in the inhibition and dispersion of *Acinetobacter baumannii*, *Pseudomonas aeruginosa* and *Staphylococcus aureus* biofilms and resensitization of these and other multidrug-resistant bacteria to antibiotics [16,21,27,28,29,30,31,32,33]. A second mechanism highlighted by our recent studies in *M. tuberculosis* and *M. smegmatis* relates to the effect of certain 2-AIs on membrane bioenergetics and the proton motive force (PMF) [19,34]. Given the known importance of membrane-mediated anaerobic metabolism in the maintenance of bacterial biofilms [35], this effect of 2-AIs is thought to be the primary driver of their antibiofilm activity in these mycobacterial species. Another report in *A. baumannii* also highlighted the ability of certain 2-AI derivatives to permeabilize bacterial membranes [36].

The ability of AB-2-29 to permeabilize the plasma membrane of *Mabs* subsp. *abscessus* ATCC 19977 was first tested using the LIVE/DEAD BacLight assay, which is based on the nucleic-acid-specific viability dyes propidium iodide and SYTO9. While 0.2 and 0.5% SDS had a dramatic permeabilization effect on the bacilli after one hour of incubation at 37 °C, AB-2-29 failed to show any such effect at its IC_50_ value (20 μM) (Figure S2).

The ability of AB-2-29 to dissipate the transmembrane potential (ΔΨ), the transmembrane electrochemical proton gradient (ΔpH) or both components of the PMF in *M. abscessus* was next tested using whole-cell-based and cell-free assays. Impact on the ΔΨ and ΔpH of intact *Mabs* ATCC 19977 bacilli grown in SCFM was determined by labeling with 3,3′-diethyloxacarbocyanine iodide (DiOC_2_(3)) and 5-chloromethyl-fluorescein diacetate (CMFDA), respectively. As shown in Figure S3, treating the bacilli with 5 or 20 μM of AB-2-29 for 4 h had no significant impact on either the intracellular pH or the ΔΨ of *M. abscessus*. A slight but significant effect on ΔΨ (but not on intracellular pH) only manifested at the highest concentration of AB-2-29 tested (100 μM; i.e., 5 times its antibiofilm IC_50_ value). Consistent with these results, AB-2-29 also failed to dissipate the ΔpH of *Mabs* inverted membrane vesicles in a succinate-driven proton translocation assay with the fluorescent substrate ACMA (Figure S4). In conclusion, at concentrations where biofilm formation was inhibited, AB-2-29 did not dissipate the PMF of *M. abscessus*.

### 2.3. Alteration of M. abscessus Response to Zinc Starvation by AB-2-29

As an unbiased approach to gain insight into the mechanism of action of AB-2-29, we next turned to RNA sequencing (RNAseq) to determine the changes undergone by the transcriptional profile of *Mabs* upon exposure to AB-2-29. Duplicate samples of exponentially growing *Mabs* ATCC 19977 cells in SCFM were exposed to 20 μM of AB-2-29 or 0.2% DMSO control for 3 or 24 h at 37 °C with shaking. A comparison of the transcriptional profiles of DMSO- and AB-2-29-treated bacilli at the 3 and 24 h time points was next conducted, and the list of differentially expressed (DE) genes (log_2_ fold-change > 2 with a false discovery rate adjusted *p* < 0.05) is presented in Table S1.

**Figure 2:**
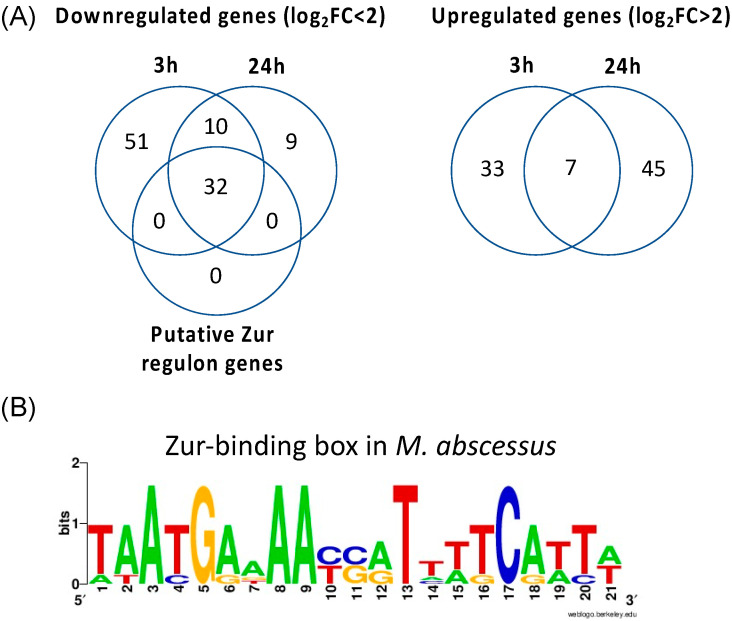
**Transcriptional response of *M. abscessus* to AB-2-29 exposure.** (**A**) Venn diagrams showing the number of genes expressed at a significantly higher or lower level in *Mabs* ATCC19977 upon exposure to 20 μM AB-2-29 for 3 and 24 h compared to DMSO-treated bacilli, and the number of genes among these that are predicted to belong to the *M. abscessus* Zur regulon. The complete list of these genes is provided in Table S1. (**B**) Consensus sequence logo for predicted *M. abscessus* Zur-binding sites. The *Mabs* ATCC 19977 genome was scanned using the Pattern Locator online software [40] for the presence of putative *Mycobacteriaceae* Zur-binding sites, as defined by Mikhaylina et al. [37] (TRWYGRNAAYSRTNNNCRWYW), in intergenic regions and allowing for up to one mismatch. The search retrieved six binding sites potentially regulating 32 genes, which we defined as Zur regulon genes. The sequence logo for the consensus Zur-binding motif in *Mabs* was constructed using WebLogo [41].

This analysis revealed 40 and 52 upregulated genes and 93 and 51 downregulated genes when comparing DMSO- versus AB-2-29-treated cells after 3 h and 24 h, respectively. A very clear pattern that emerged was a strong (>2.4 to 10.5 log_2_-fold) downregulation of genes required for adaptation to zinc starvation in the AB-2-29 treatment groups, both at the 3 and 24 h time points. Indeed, at both time points, all 32 predicted Zur regulon genes of *M. abscessus* were expressed at a significantly lower level in the AB-2-29-treated bacilli (Table 2; Figure 2). Other DE genes at both time points were few and essentially encompassed genes involved in lysine and cobalamin biosynthesis, glyoxylase/bleomycin resistance, a β-lactamase gene and genes encoding a number of hypothetical proteins of unknown function. RNAseq analyses otherwise failed to reveal any two-component system regulators among the DE genes, suggesting that AB-2-29 may act differently from other prototypical 2-AIs in inhibiting *M. abscessus* biofilm formation.

The “zinc uptake regulator” Zur is the most widespread zinc-responsive transcriptional factor in prokaryotes [37]. In mycobacteria as in most other prokaryotes, Zur transcriptional regulators act as repressors when Zn^2+^ is not limiting in the culture medium. Under these conditions, Zn^2+^ ions become bound to Zur, enabling the protein to bind a *zur-box* in the promoter region of a number of genes, which results in the blockage of the binding site for the RNA polymerase transcription initiation complex. Under low-zinc conditions, Zur dissociates from the *zur-box* derepressing the transcription of a variety of genes involved in Zn^2+^ uptake and the production of zinc-independent enzymes (including zinc-independent ribosomal proteins), among others [37,38,39]. In our study, the strong expression of Zur-regulated genes in DMSO-treated cells was not unexpected given the absence of added zinc in the SCFM medium used to culture *Mabs* ATCC 19977 (Table S1; Table 2; Figure 2). The fact that the level of expression of these genes considerably decreased in the AB-2-29-treated bacilli was thus indicative of either the presence of zinc brought into the medium by the inhibitor itself or of the ability of the inhibitor to block the Zur repressor in a DNA-binding (i.e., repressing) conformation, even in the absence of zinc in the medium.

### 2.4. AB-2-29 Binds Zinc

**Figure 3:**
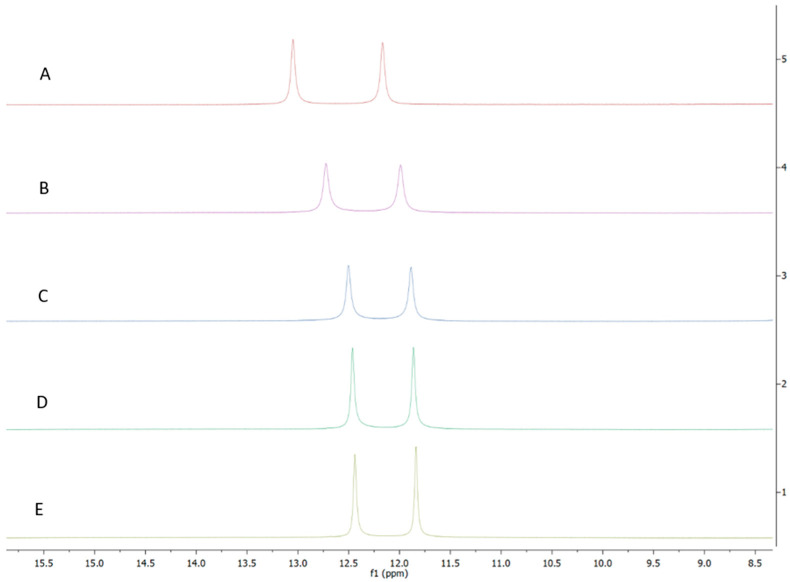
**^1^H NMR spectra of AB-2-29 with (A) 0.0 (B) 0.5 (C) 1.0 (D) 2.0 (E) 4.0 equivalents of zinc.** The shifting of the NH_2_ peaks (between 11.5 and 13.0 ppm) with increased equivalents of zinc implies complexation between AB-2-29 and zinc.

To differentiate between these two hypotheses, we first sought to determine whether AB-2-29 bound zinc, especially since there was precedence for compounds based on the related 2-aminobenzimidazole scaffold inhibiting *Staphylococcus aureus* biofilms through their ability to chelate zinc from the medium [42,43]. Atomic absorption spectroscopy and NMR-based [44] analyses both converged to indicate that AB-2-29 binds zinc (Figure 3). In contrast, the inhibitor did not bind Fe^2+^ (Figure S5). Per atomic absorption spectroscopy analysis, 0.126 moles of Zn^2+^ came with every one mole of the AB-2-29 batch used in our experiments.

Since Zn^2+^ ions are naturally absent from SCFM, where *M. abscessus* forms abundant biofilms, one can exclude that AB-2-29 prevents biofilm formation by acting as a zinc chelator. Likewise, we exclude that the amount of zinc that comes with AB-2-29 (2.52 μM of zinc at its IC_50_ value of 20 μM) is responsible for the observed antibiofilm activity of this compound, since we previously established that much higher concentrations of zinc were required to inhibit *M. abscessus* biofilm formation in SCFM (IC_50_ of zinc~150 μM against *Mabs* NJH12) [11]. The fact that AB-2-29 comes with small quantities of zinc, however, explains the repression of *zur* regulon genes in AB-2-29-treated cultures.

### 2.5. Potentiation of the Biofilm Inhibitory Properties of AB-2-29 by Zinc

**Figure 4:**
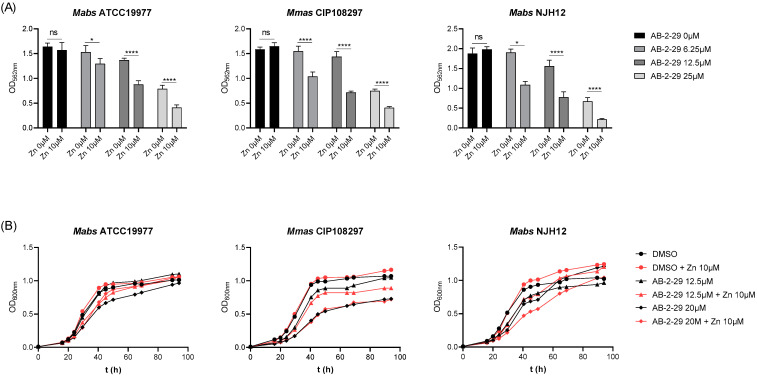
**Potentiation of the antibiofilm activity of AB-2-29 by zinc**. (**A**) Biofilm formation by *Mabs* ATCC 19977, *Mabs* NJH12 and *Mmas* CIP108297 in SCFM after 5 days of incubation in the presence or absence of AB-2-29 (0, 6.25, 12.5 or 20 μM) and zinc (0 or 10 μM). The results presented are the means (±SD) of sextuplicate wells and are representative of at least two independent experiments. Asterisks denote statistically significant differences between biofilm inhibitor treatment with and without zinc (* *p* < 0.05 and **** *p* < 0.00005); ns: not significant. (**B**) Growth of *Mabs* ATCC 19977, *Mabs* NJH12 and *Mmas* CIP108297 in SCFM in the presence or absence of AB-2-29 (0, 6.25, 12.5 or 20 μM) and zinc (0 or 10 μM). The results presented are representative of at least two independent experiments.

Given the zinc-binding properties of AB-2-29, we next sought to determine how the presence of zinc in the medium affected the activity of the inhibitor. To this end, biofilm assays were repeated in the absence or presence of different concentrations of ZnSO_4_ in SCFM. The results, which are presented in Figure 4A, showed a striking potentiation of the antibiofilm activity of AB-2-29 against three different *Mabs* and *Mmas* strains in the presence of zinc. Since the same treatments did not noticeably impact bacterial growth (with the exception of *Mmas* CIP108297 whose growth rate was slightly reduced in the presence of 20 μM AB-2-29), one can exclude that the reduction in biofilm formation observed in the AB-2-29 +/− Zn^2+^-treated groups was in fact the result of growth inhibition (Figure 4B).

## 3. Discussion

A novel inhibitor of *M. abscessus* biofilm formation has been identified that not only binds to zinc but whose activity also increases in the presence of zinc. The precise mechanism(s) underlying the antibiofilm activity of AB-2-29 and its potentiation by zinc remain(s) to be determined. Unlike previously reported 2-AI compounds and related scaffolds with antibiofilm activity against other Gram-negative or Gram-positive bacteria [16,19,21,27,28,29,30,31,32,33,34,36], AB-2-29 does not appear to act by dissipating the proton motive force or by permeabilizing the plasma membrane of *M. abscessus*. Likewise, no clear evidence could be derived from our RNAseq studies that AB-2-29 interfered with a particular two-component regulatory system in *Mabs* ATCC 19977, although one cannot exclude that such a mechanism is at play. Although the amount of zinc brought by AB-2-29 to the culture medium is not sufficient to explain, by itself, the antibiofilm activity of this compound, one may speculate that the complexation of zinc by AB-2-29 facilitates the penetration of the inhibitor inside the cells. Alternatively, the repression of Zur regulon genes caused by AB-2-29 may contribute, at least in part, to its activity. Indeed, the zinc-independent ribosomal proteins S18, S14, L33 and L28 have been involved in biofilm formation in *M. smegmatis* [45]. Moreover, we note that a number of genes that are downregulated upon exposure to AB-2-29 (*MAB_1044c*, *MAB_1046c*, *MAB_2204*, *MAB_2706c*, *MAB_3438* and the Zur-regulated Mce operon encoded by the gene cluster encompassing *MAB_1680* to *MAB_1701*) were found to be upregulated in *M. abscessus* during biofilm development [11]. Clearly, further studies are needed to assess the relative contribution of these genes, either individually or combined, to the biofilm-forming capacity of *M. abscessus*. The importance of such studies resides in their potential to lead to better targeted strategies to inhibit *M. abscessus* biofilm formation during infection.

Interestingly, AB-2-29 is not the first example of a metal complex displaying antibiofilm activity against *M. abscessus*. Indeed, metal complexes made of gold, silver, copper or cadmium-containing sulfonamides have recently been shown to display similar properties against a variety of NTM [46,47]. Given the relatively high concentration of zinc, magnesium, calcium and iron found in the sputum of persons with CF and non-CF bronchiectasis [48], this observation provides a basis for the development of innovative therapeutics and adjunct therapeutics directed against difficult-to-cure NTM pulmonary infections.

## 4. Materials and Methods

### 4.1. Bacterial Strains and Culture Media

Reference strains *Mabs* ATCC 19977 and *Mmas* CIP 108297 were obtained from the ATCC and CIP collections, respectively. *M. abscessus* subsp. *abscessus* and *M. abscessus* subsp. *massiliense* clinical isolates, NJH9, NJH12 and NJH18 were from persons with CF at National Jewish Health (Denver, CO, USA) [11]. *M. abscessus* subsp. *massiliense* clinical isolate 1239 was from a person with CF at the Royal Papworth Hospital, Cambridge, UK [26]. *M. abscessus* strains were grown under agitation at 37 °C in Middlebrook 7H9 medium supplemented with 10% albumin-dextrose-catalase (ADC) (BD Sciences) and 0.05% Tween 80, in SCFM [11] or on Middlebrook 7H11 agar supplemented with 10% oleic acid-albumin-dextrose-catalase (OADC) (BD Sciences). To assess the impact of zinc on growth and biofilm formation, ZnSO_4_ was added at different concentrations to SCFM.

### 4.2. Biofilm Assay

*M. abscessus* submerged biofilms were formed in 96-well (polystyrene, flat bottom) poly-D-lysine-coated plates in 200 μL of SCFM and monitored by crystal violet staining as described [11].

### 4.3. Drug-Susceptibility Testing

The minimum inhibitory concentrations (MICs) of 2-AI-based small-molecule compounds against *Mabs* ATCC 19977 and *Mmas* CIP108297 grown planktonically were determined in SCFM in a total volume of 200 μL in 96-well microtiter plates. Cultures grown to early log phase were diluted to a final concentration of 10^6^ CFU mL^−1^ and incubated in the presence of serial dilutions of the compounds for 5 days at 37 °C. MICs were determined as the lowest concentration of 2-AI-based compound, showing no visible growth.

### 4.4. Membrane Permeabilization Assay

Plasma membrane permeabilization by AB-2-29 and SDS was measured with the LIVE/DEAD BacLight kit (Thermo Fisher, Waltham, MA, USA). The fluorescence of *Mabs* ATCC 19977 bacilli, either treated with DMSO (0.2% final concentration), SDS (0.2 and 0.5%) or AB-2-29 (5 and 20 μM in 0.2% DMSO final concentration) for 1 h at 37 °C, followed by incubation with LIVE/DEAD BacLight solution for 15 min at room temperature, was measured using an excitation wavelength of 485 nm and emission wavelengths of 535 nm (green) and 615 nm (red).

### 4.5. Membrane Potential and Electrochemical Proton Gradient Measurements in Intact M. abscessus Bacilli

The effects of AB-2-29 on the transmembrane potential (ΔΨ) and transmembrane electrochemical proton gradient (ΔpH) of intact *Mabs* ATCC 19977 cells were determined by fluorescence quenching of the membrane potential-sensitive probe 3-3′ Diethyloxacarbocyanine iodide (DiOC_2_(3)) (Thermo Fisher, Waltham, MA, USA) and the pH-sensitive probe 5-chloromethyl-fluorescein diacetate (CMFDA) (Thermo Fisher, Waltham, MA, USA), respectively, as described [49].

### 4.6. Assay for Succinate-Driven Proton Translocation into M. abscessus Inverted Membrane Vesicles (IMVs)

Succinate-driven proton translocation assays with the fluorescent substrate ACMA were conducted as previously described [49] to determine the effect of AB-2-29 on the electrochemical proton gradient of *Mabs* ATCC 19977 IMVs. IMVs (0.2 mg mL^−1^ membrane proteins) were preincubated at 37 °C in 10 mM HEPES-KOH pH 7.5, 100 mM KCl, 5 mM MgCl_2_ containing 20 μM ACMA, and the baseline was monitored for 10 min with a Victor X5 fluorescence spectrophotometer (PerkinElmer, Waltham, MA, USA). The reaction was then initiated by adding 5 mM succinate. Upon stabilization of the signal, control compounds (nigericin), AB-2-29 or diluent (0.2% DMSO) were added, and proton translocation was monitored fluorometrically. The excitation and emission wavelengths were 419 nm and 483 nm, respectively.

### 4.7. RNA Extraction, Reverse Transcription and RNAseq

Two independent cultures of DMSO (0.2%)- or AB-2-29 (20 μM)-treated *Mabs* ATCC 19977 were used for transcriptomics analyses. RNA extraction, reverse transcription reactions, RNAseq library preparation and RNAseq data analysis were conducted as described previously [11].

### 4.8. Atomic Absorption Spectroscopy

AB-2-29 was resuspended in DMSO at a final concentration of 10 mM, and 90 μL of sample was mixed with 200 μL HNO_3_ and incubated for 1 h at 80 °C and overnight at 20 °C. Digestions were concluded after addition of 60 μL of 30% H_2_O_2_ and dilution to 2 mL with water. Samples were dried overnight at 65 °C and weighed on a 5-place scale. Then, samples were dry-washed overnight at 600 °C, dissolved in 3.6 N HNO_3_, diluted and analyzed using an atomic absorption spectrophotometer Model 240AA (Agilent) with reference standards for each metal. DMSO alone was included as control.

### 4.9. Chemical Synthesis

4-(4-propoxyphenyl)-1H-imidazol-2-amine hydrochloride (AB-2-29) was synthesized as previously reported [22].

### 4.10. Zinc and Iron Binding Studies

DMSO-*d*6 was purchased from Sigma Aldrich. Fe(II)sulfate-heptahydrate and Zn(II)Cl_2_ were purchased from Acros Organics. For zinc binding studies, 4-(4-propoxyphenyl)-1H-imidazol-2-amine hydrochloride was dissolved in 500 μL of DMSO-*d*6 dosed with the appropriate equivalents of anhydrous Zn(II)Cl_2_. ^1^H NMR spectra were obtained on a Bruker Avance spectrometer (400 MHz) at ambient temperature. For iron binding studies, 5 μL of a 10 mM stock of 4-(4-propoxyphenyl)-1H-imidazol-2-amine hydrochloride in DMSO was pipetted into 1 mL of the appropriate aqueous solution of Fe(II)SO_4_ heptahydrate. The samples were vortexed thoroughly and left at ambient temperature for 20 min. The UV spectra were obtained on a SynergyHTX (BioTek, Winooski, VT, USA) multimode plate reader using a Take3 plate and BioCell (BioTek), scanning from 200 to 800 nM.

## Figures and Tables

**Table 1:** IC_50_ values for the inhibition of *M. abscessus* biofilms. Biofilm assays and MIC determinations were repeated at least two times. n.d., not determined. MICs were determined against all isolates for which biofilm assays were run. MIC values were the same for all isolates unless otherwise indicated.

Compound	MIC (M)	IC_50_ (M)					
		*Mmas* CIP 108297	*Mabs* ATCC 19977	*Mabs* NJH12	*Mmas* 1239	*Mabs* NJH9	*Mmas* NJH18
EL-05-047 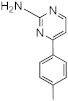	>100	>100	n.d.	50–100	n.d.	50–100	100
2B8 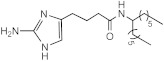	25	12.5–25	n.d.	25	n.d.	12.5–25	25
SEM-002-004 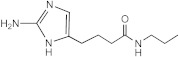	>50	>200	n.d.	n.d.	n.d.	n.d.	n.d.
SEM-001-075 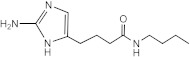	>50	>200	n.d.	n.d.	n.d.	n.d.	n.d.
SEM-001-073 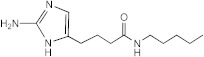	>50	>200	n.d.	n.d.	n.d.	n.d.	n.d.
SEM-001-078 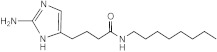	100–200	50	n.d.	n.d.	n.d.	n.d.	n.d.
SEM-002-003 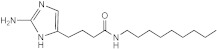	50–100 (*Mmas* CIP 108297 and *Mabs* ATCC 19977) 50 (*Mabs* NJH9, *Mabs* NJH12 and *Mmas* NJH18)	25	n.d.	12.5–25	n.d.	12.5–25	50
RA10 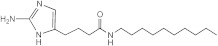	>50	25	n.d.	n.d.	n.d.	n.d.	n.d.
RA12 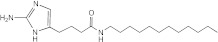	50	>50	n.d.	n.d.	n.d.	n.d.	n.d.
RA13 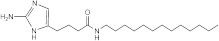	>50	50	n.d.	n.d.	n.d.	n.d.	n.d.
SEM-001-034 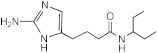	>50	>200	n.d.	n.d.	n.d.	n.d.	n.d.
SEM-001-044 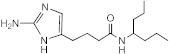	>50	>200	n.d.	n.d.	n.d.	n.d.	n.d.
SEM-001-046 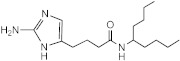	100–200	50	n.d.	n.d.	n.d.	n.d.	n.d.
SEM-001-056 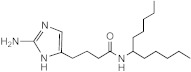	50	12.5–25	12.5–25	25	25	12.5–25	25
SEM-001-049 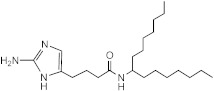	12.5–25	25	n.d.	n.d.	n.d.	n.d.	n.d.
SEM-001-050 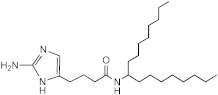	50 (*Mabs* NJH9, *Mabs* NJH12, *Mmas* NJH18, *Mmas* CIP108297) 100 (*Mabs* ATCC 19977)	12.5–25	n.d.	12.5–25	n.d.	12.5–25	12.5–25
SEM-001-057 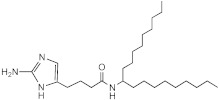	50–100	50	n.d.	n.d.	n.d.	n.d.	n.d.
VN03-049 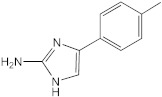	50–100	25	n.d.	n.d.	n.d.	n.d.	n.d.
VN03-063 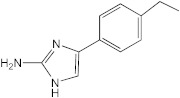	25–50	25	n.d.	n.d.	n.d.	n.d.	n.d.
VN03-074 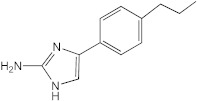	50–100	25–50	n.d.	n.d.	n.d.	n.d.	n.d.
4C3 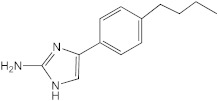	25–50	12.5	n.d.	n.d.	n.d.	n.d.	n.d.
4B10 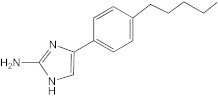	12.5–25	12.5	n.d.	n.d.	n.d.	n.d.	n.d.
4C2 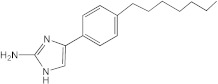	50	12.5–25	n.d.	n.d.	n.d.	n.d.	n.d.
VN03-064 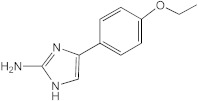	25	25	n.d.	n.d.	n.d.	n.d.	n.d.
AB-2-29 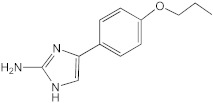	>100	25	25	12.5–25	50	12.5	25
AB-2-24 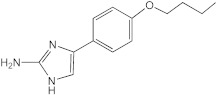	64	12.5	25–50	12.5	25	12.5–25	12.5–25
AB-2-26 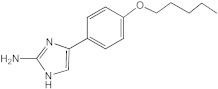	64	12.5–25	25–50	12.5–25	25	6.25–12.5	12.5–25
7.079 (meridianin) 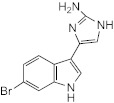	>100	100	100	50	n.d.	50–100	100
7.025 (meridianin) 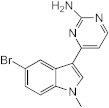	>100	>100	100	50–100	n.d.	>100	50–100
8.001 (meridianin) 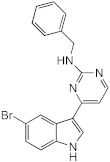	>100	>100	>100	>100	n.d.	>100	>100

**Table 2:** **List of Zur regulon genes that were expressed at higher or lower levels upon treatment with AB-2-29 compared to DMSO control**. Differentially expressed genes upon treatment with 20 μM AB-2-29 for 3 or 24 h were defined as ≥ 2 log_2_ fold-change in expression compared to cells treated with 0.2% DMSO for the same amount of time, with a false discovery rate adjusted *p*-value (padj) < 0.05. Genes harboring a *zur-box* in their promoter (see Figure 2B) are marked with an asterisk. Similarly colored genes denote gene clusters likely to be cotranscribed. MAB_0331c, MAB_0332c, MAB_0333c, MAB_0334c and MAB_0336 are Zn-independent alternative ribosomal proteins. MAB_0335 is likely to be involved in cobalamin biosynthesis. *MAB_0575c-MAB_0576c-MAB_0577c* encode a putative zinc importer of the ABC-transporter family. The operon encompassing genes *MAB_1680* to *MAB_1701* encodes putative Zn-siderophore biosynthesis and transport proteins, including a putative ABC-transporter and an MCE family transporter.

Gene	Description	Putative Function	Log_2_ Fold-Change	
			AB-2-29 vs. Ctrl 3 h	AB-2-29 vs. Ctrl 24 h
*MAB_0331c*	30S ribosomal protein S18 RpsR2	Zn-independent ribosomal proteins	−8.04	−9.31
*MAB_0332c*	30S ribosomal protein S14 RpsN2		−7.76	−8.93
*MAB_0333c*	50S ribosomal protein L33 RpmG1		−9.72	−6.26
*MAB_0334c* *	50S ribosomal protein L28 RpmB2		−9.30	−7.83
*MAB_0335* *	Probable cobalamin synthesis protein	Cobalamin biosynthesis	−7.95	−10.46
*MAB_0336*	50S ribosomal protein L31 type B	Zn-independent ribosomal protein	−8.52	−9.38
*MAB_0575c*	Putative ABC-transporter transmembrane protein	ZnuABC transporter (Zn import)	−2.91	−2.39
*MAB_0576c*	Putative ABC-transporter ATP-binding protein		−4.05	−2.89
*MAB_0577c* *	Putative ABC-transporter solute binding protein		−5.54	−5.40
*MAB_0809c* *	Conserved hypothetical PPE family protein	Unknown	−5.77	−4.66
*MAB_1680* *	Hypothetical protein	Zn-siderophore biosynthesis and transport	−9.76	−8.14
*MAB_1681*	Hypothetical protein		−9.84	−7.80
*MAB_1682*	Probable NAD-dependent epimerase/dehydratase		−9.04	−8.69
*MAB_1683*	Putative fatty acid desaturase		−10.30	−7.96
*MAB_1684*	Diaminobutyrate-−2-oxoglutarate aminotransferase		−9.48	−8.31
*MAB_1685*	Putative decarboxylase		−7.35	−7.01
*MAB_1686*	Hypothetical protein		−8.48	−7.31
*MAB_1687*	Hypothetical protein		−8.09	−8.20
*MAB_1688*	Hypothetical protein		−7.85	−7.90
*MAB_1689*	Probable ABC-transporter ATP-binding subunit DrrA		−7.99	−7.48
*MAB_1690*	Putative ABC-transporter transmembrane protein		−8.82	−6.46
*MAB_1691*	Hypothetical protein		−9.86	−8.31
*MAB_1692*	Putative polyketide synthase Pks16/acyl-CoA synthetase		−7.95	−7.05
*MAB_1693*	Conserved hypothetical protein (YrbE family?)		−8.46	−7.52
*MAB_1694*	Putative YrbE family protein		−8.65	−6.58
*MAB_1695*	Putative Mce family protein		−8.60	−5.87
*MAB_1696*	Putative Mce family protein		−7.56	−6.09
*MAB_1697*	Putative Mce family protein		−7.06	−6.29
*MAB_1698*	Putative Mce family protein		−7.13	−5.57
*MAB_1699*	Putative Mce family protein		−7.33	−4.90
*MAB_1700*	Putative Mce family protein		−6.82	−5.02
*MAB_1701*	Hypothetical protein		−5.82	−3.61

## Data Availability

The sequencing data described in this publication have been submitted to the NCBI gene expression omnibus (GEO) under BioProject No. PRJNA658400.

## Supplementary Materials

The following are available online at https://www.mdpi.com/article/10.3390/ijms23062950/s1.
