# Supplementary material for: 2-Aminoimidazoles Inhibit Mycobacterium abscessus Biofilms in a Zinc-Dependent Manner
Source: Int J Mol Sci. 2022 Mar 9;23(6):2950. doi: 10.3390/ijms23062950 (PMC8951752; doi:10.3390/ijms23062950)

Supplementary Materials

Table S1 [see separate Excel file]: RNA-seq dataset of differentially expressed genes in all comparisons.

Differentially expressed (DE) genes in *Mabs* ATCC 19977 cultured in the presence of 20 μM AB-2-29 for 3 and 24 hours were defined as ≥2 Log_2 fold-change in expression compared to cells treated with 0.2% DMSO for the same amount of time, with a false discovery rate adjusted *p*-value (padj) <0.05.

<u>Figure S1</u>: Effect of AB-2-29 on *M. abscessus* biofilm dispersal.

Four-day-old *Mabs* ATCC 19977 biofilms were washed with PBS and AB-2-29 (or 0.2% DMSO as control) in PBS were added at the indicated concentrations (in µM). The plate was incubated for another 24 h at 37°C at which point biofilms were quantified by crystal violet staining. The results presented are the means (± SD) of quadruplicate wells.

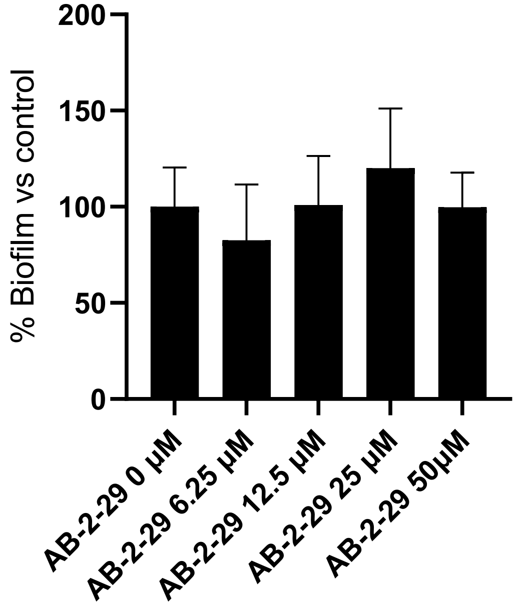

<u>Figure S2</u>: Plasma membrane permeabilization by LIVE/DEAD BacLight after exposure of *Mabs* ATCC 19977 to AB-2-29.

The values represent the averages and standard deviations of measurements performed on three independent bacterial suspensions after exposure to AB-2-29, SDS or DMSO. Asterisks denote statistically significant differences between DMSO controls and SDS- or AB-2-29-treated cells per the Student's *t*-test (***$p < 0.0005$).

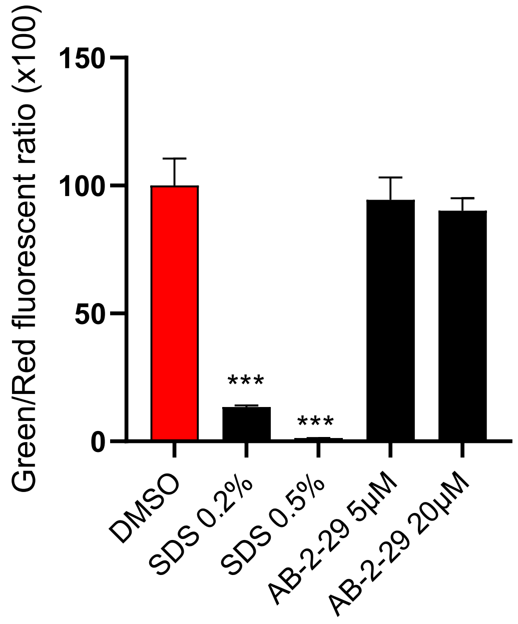

<u>Figure S3</u>: Effect of AB-2-29 on the electrochemical proton gradient (ΔpH) and membrane potential (ΔΨ) of *Mabs* ATCC 19977.

The inner bacterial pH **(A)** and ΔΨ **(B)** of *Mabs* ATCC 19977 cells grown in the presence of 5, 20 and 100 μM of AB-2-29, 0.2% DMSO or PMF dissipators (valinomycin, nigericin and CCCP; each used at 20 μM) were determined. The values represent the averages and standard deviations of measurements performed on three independent bacterial suspensions after exposure to the various compounds or the DMSO solvent for 30 minutes at 37°C. Results are representative of two independent tests. Asterisks denote statistically significant differences compound-treated and DMSO-treated cells per the Student's *t*-test (*$p < 0.05$; ** $p < 0.005$).

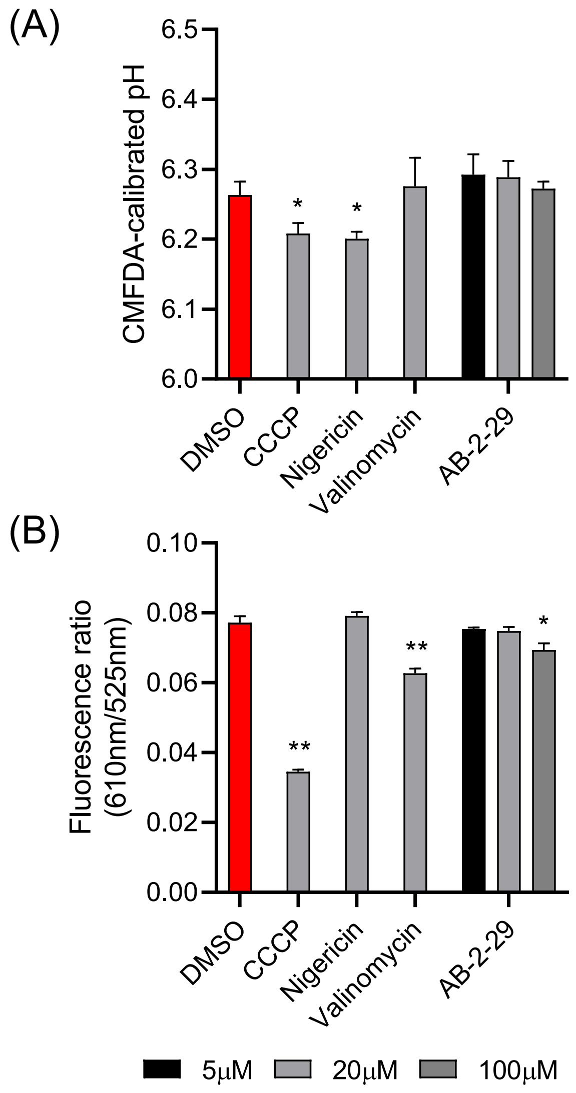

<u>Figure S4</u>: Effect of AB-2-29 on the electrochemical proton gradient (ΔpH) of *Mabs* ATCC 19977 inverted membrane vesicles (IMVs).

ACMA fluorescence assay using *Mabs* ATCC 19977 IMVs. The reactions were initiated by adding 5 mM succinate. Upon stabilization of the signal, control compound (20 µM CCCP), AB-2-29 (5, 20 or 100 µM) or diluent (0.2% DMSO) were added and proton translocation monitored fluorometrically. Shown are the averages and standard deviations (error bars) of triplicate measurements. Addition of CCCP results in an increase of fluorescence intensity indicative of ΔpH collapse.

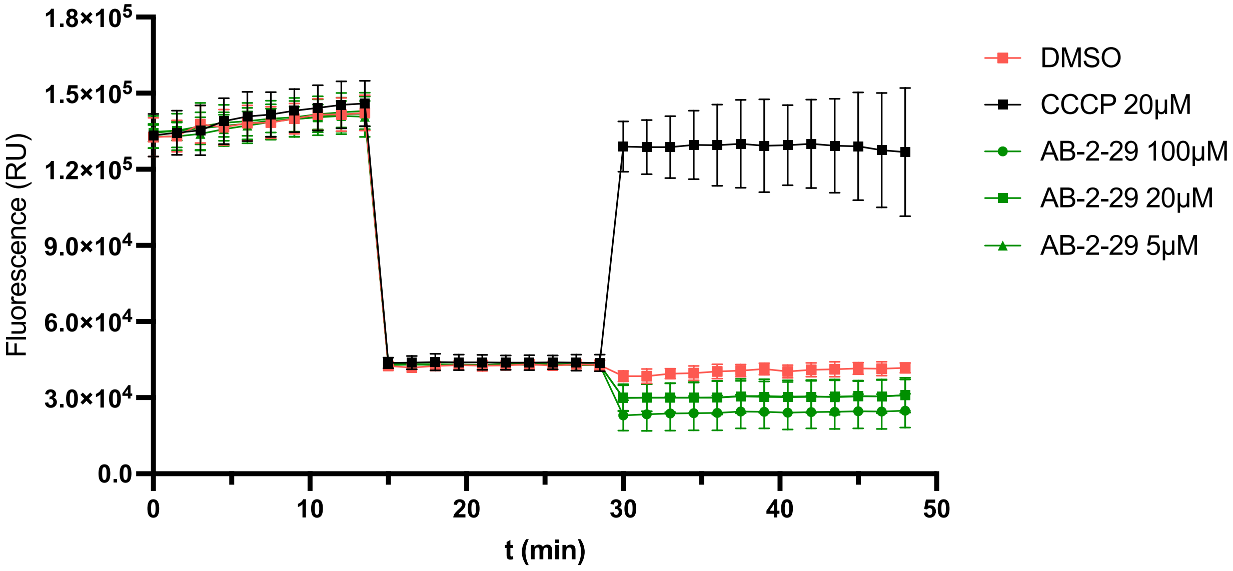

<u>Figure S5</u>: UV/Vis spectra of AB-2-29 with increasing concentrations of $Fe^{II}(SO_4)$. Absence of any effect on the UV spectrum with addition of iron suggests that AB-2-29 does not bind Fe^{2+}.

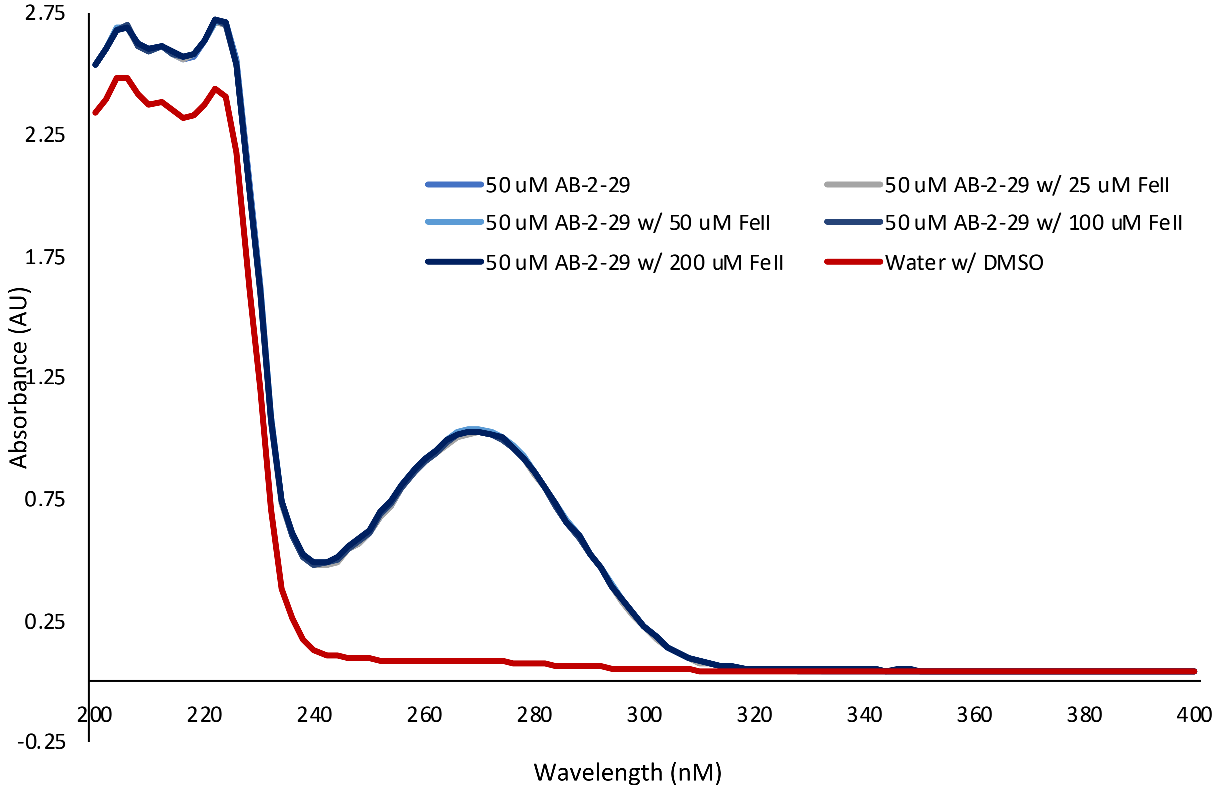

Supplement: Supplementary file 1 [file ijms-23-02950-s001.zip › Supplementary Materials.pdf]
